# Plant produced endotoxin binding recombinant proteins effectively remove endotoxins from protein samples

**DOI:** 10.1038/s41598-022-20776-6

**Published:** 2022-09-30

**Authors:** Md Rezaul Islam Khan, Muthamilselvan Thangarasu, Hyangju Kang, Inhwan Hwang

**Affiliations:** 1 Department of Life Sciences, Pohang University of Science and Technology, Pohang, 37673 Korea; 2 R&D Center, BioApplications Inc., Technopark Complex, Pohang, 37668 Korea

**Keywords:** Biological techniques, Biotechnology, Plant sciences

## Abstract

Lipopolysaccharides (LPS) are highly toxic compounds, even at a trace amount. When recombinant proteins are produced in *E*. *coli*, it is inevitable that LPS contaminates. However, LPS removal is still technically challenging and costly due to the high degree of solubility in a wide range of solvents. In this study, we explored the possibility of using the N-terminal region containing cysteine-rich, EGF-like, and sushi1–3 domains (CES3) of Factor C from the horseshoe crab *Carcinoscorpius rotundicauda* to develop a platform to remove LPS from recombinant proteins. We expressed CES3 as part of a recombinant protein, BiP:NT:CBM3:SUMO:CES3:His:HDEL, in *Nicotiana benthamiana* and found that purified or microcrystalline cellulose (MCC) bead-immobilised CES3 showed strong binding to LPS-containing *E*. *coli*. To produce CES3:CBM3 in an LPS-free environment, we generated *Arabidopsis* transgenic plants harbouring a recombinant gene, *BiP:NT:SUMO:CES3:CBM3:HDEL*, and found that transgenic plants mainly produce CES3:CBM3:His:HDEL, a truncated version of BiP:NT:SUMO:CES3:CBM3:HDEL via endogenous protease-mediated proteolytic processing in vivo. CES3:CBM3:HDEL purified from *Arabidopsis* plant extracts and immobilised onto MCC beads removed LPS contamination from protein samples. We propose that the CES3:CBM3 fusion protein produced in plants and immobilised on MCC beads can be a robust and easy platform for LPS removal from recombinant proteins.

## Introduction

Recombinant proteins are in great demand for pharmaceutical use. Various organisms have been developed as platforms to produce recombinant proteins. Of these platforms, *E*. *coli* is still favourable due to fast growth, use of simple growth medium, availability of a variety of genetic tools, high yield of protein production, and a great deal of experience in the production of vastly diverse proteins. Indeed, *E*. *coli* is the host of 30–40% of approved therapeutic proteins today^1–3^. Despite this success, *E*. *coli* as a host for recombinant protein production still has a serious limitation—endotoxin contamination^3–8^.

Endotoxin is a domain of LPS, a major component of the outer membrane of Gram-negative bacteria. LPS consists of three domains: a hydrophobic domain known as lipid A (or endotoxin), a nonrepeating core oligosaccharide, and a distal polysaccharide (or O-antigen)^9^. Lipid A (endotoxin) of LPS is responsible for toxicity by triggering proinflammatory cytokine production^10,11^. The presence of a trace amount of endotoxin, as low as 1 ng of endotoxin per kg body weight, can cause a pyrogenic reaction^12,13^. However, it is not easy to remove due to its high stability and resistance to heat and a range of pH. Moreover, given its chemical nature, endotoxins can interact with both hydrophobic and hydrophilic materials under diverse physiological conditions. Various methods have been developed and widely applied to remove endotoxins from pharmaceutical products^8,14–20^. These include filtration, hydrophilic–hydrophobic phase separation, and ion-exchange chromatography^14,17,21,22^. However, these methods are limited by one or more aspects, such as high cost, low productivity, low efficiency, low protein recovery, effect on protein stability, or contamination of undesirable chemicals. Recently, the focus of endotoxin removal has shifted to affinity absorbents that include dimethylaminoethanol, poly-1-lysine, poly(ethyleneimine) (pEI), histamine, histidine, diaminohistidine, polyvinyl alcohol, chitosan, and polymixin B. These ligands were immobilised on the membranes of cellulose, cellulose acetate, nylon, or polyvinyl acetate^8^. Recently, nanoparticles (NPs) have been used to immobilise ligands, such as poly-ε-caprolactone (PCL)^8^.

The haemocyte of horseshoe crabs (*Carcinoscorpius rotundicauda*) is attributed to its extraordinary ability to coagulate Gram-negative bacteria by a cascade of pathways initiated by a protein called Factor C, a serine protease zymogen, the main component of the LAL endotoxin test kit^23,24^. Factor C is composed of an N-terminal signal peptide (1–25), a heavy chain (26–690), and a light chain (691–1019). The heavy chain contains multiple domains. Of these domains, the cysteine-rich domain, or sushi domain, is proposed to bind to LPS^25,26^. However, Koshiba et al.^27^ suggested that tripeptide, Arg36-Trp37-Arg38, at the cys-rich domain of the horseshoe crab *Tachypleus tridentatus* is essential for LPS binding and substitution of these three residues leads to loss of LPS-binding ability. The C-terminal region contains a serine protease domain. After processing, the two chains are connected via a disulphide bond. Factor C shows an extremely high affinity to LPS with 1.7 × 10^–10^ M of kd, the highest binding affinity among LPS-binding molecules. Moreover, Factor C showed positive cooperativity in LPS binding that culminates in an increase in the kd value 2.7 × 10^–12^ M^25^. LPS binding to the N-terminal region leads to the activation of Factor C, which in turn activates Factor B in the cascade of the coagulation pathway upon bacterial infection. Recombinant Factor C (rFC) has been produced in insect and animal cells for the purpose of using it to detect LPS^28,29^. Indeed, LPS test kits have now been developed using recombinant Factor C, thereby eliminating the use of haemocytes from horseshoe crabs^29^.

In this study, we investigated the possibility of using the LPS-binding ability of Factor C to develop an endotoxin removal system from protein samples. For this purpose, we expressed the N-terminal LPS binding region containing cysteine-rich, EGF-like, and three sushi domains (CES3) of Factor C as part of a recombinant protein in plants, immobilised it onto the solid surface of microcrystalline cellulose (MCC) beads using CBM3, and tested for the removal of LPS from protein samples. We showed that a polypeptide consisting of CES3 and CBM3 (CES3:CBM3) was expressed in soluble form in *Arabidopsis* transgenic plants. Moreover, CES3:CBM3 immobilised onto MCC beads effectively removed LPS from the protein samples.

## Results

### Expression of recombinant proteins containing the LPS-binding region CES3 of Factor C in *Nicotiana benthamiana*

**Figure 1:**
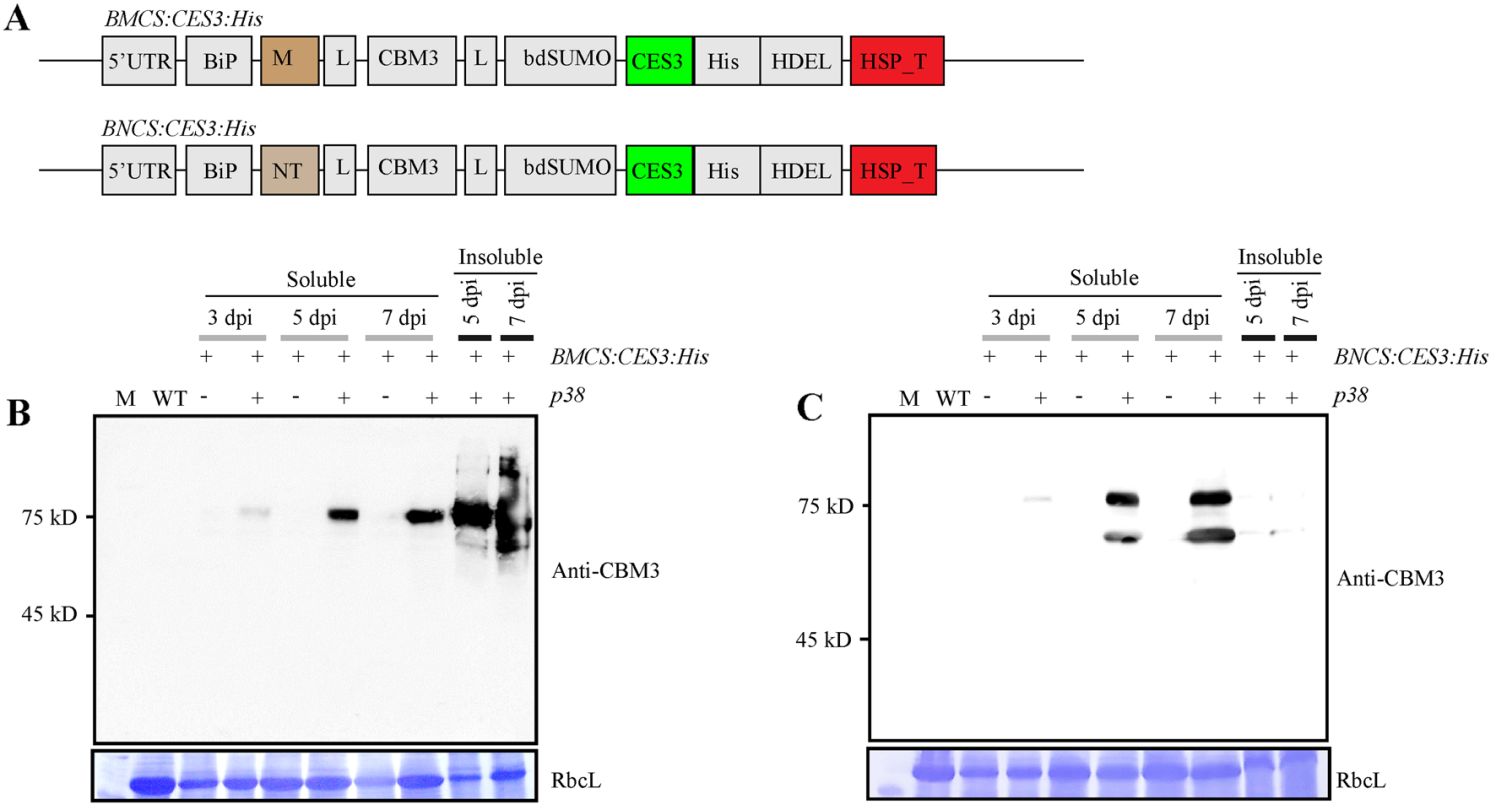
Expression of CES3-containing recombinant protein in *Nicotiana benthamiana*. (**A**) Schematic presentation of CES3-containing recombinant genes. BiP, the leader sequence of *Arabidopsis* BiP1; M, ectodomain (aa residues from Ala231 to Asp290) of human protein tyrosine phosphatase receptor type C (CD45); CBM3, cellulose-binding module 3; bdSUMO, Brachypodium SUMO; CES3, the N-terminal region containing cysteine-rich, EGF-like, and suchi 1–3 domains of Factor C from *Carcinoscorpius rotundicauda*; 5’-UTR, a translation enhancing 5’ UTR; NT-m, N-terminal region of spider silk major ampullate spidroin 1B precursor protein (NcMasp1b) from *Nephila clavipes* with mutations Asp59/Lys and Lys84/Asp. The recombinant genes *BMCS:CES3:His* and *BNCS:CES3:His* were placed under the double enhancer CaMV 35S promoter, followed by transcription terminator *Hsp-T* of *Arabidopsis Hsp18*.*2*. (**B**, **C**) Expression of *BMCS:CES3:His* and *BNCS:CES3:His*. Leaves of *N*. *benthamiana* were infiltrated with *Agrobacteria* harbouring vectors *BMCS:CES3:His* (**B**) or *BNCS:CES3:His* (**C**), together with ( +) or without (−) *Agrobacteria* harbouring *p38*. The infiltrated leaves were harvested at 3, 5, and 7 dpi. Total protein extracts of soluble proteins and cellular debris containing insoluble proteins were separated by SDS-PAGE and analysed by western blotting using an anti-CBM3 antibody. The membranes were stained with CBB, and RbcL was used as a loading control.

To explore the possibility of using the LPS-binding domain, CES3, of Factor C in a device for removing LPS in biological samples, we examined whether the N-terminal region of Factor C could be expressed as part of a recombinant protein at a high level in plants. We first used *Nicotiana benthamiana* as a host. It has been widely used as a plant for recombinant protein production^30^. Of the full-length Factor C, we focused on the production of the N-terminal region containing cysteine-rich, EGF-like, sushi1-3 domains (named CES3) as part of recombinant proteins. To express CES3, we generated a recombinant gene, *BiP:M:CBM3:SUMO:CES3:His:HDEL (BMCS:CES3:His)*, consisting of the *Arabidopsis* BiP1 leader sequence for targeting the endoplasmic reticulum (ER), translational enhancer M domain, cellulose-binding module CBM3, SUMO domain, CES3, His tag, and ER retention signal HDEL (Fig. 1A)^31^. The M domain, an extracellular domain containing multiple N-glycosylation sites from human tyrosine phosphatase receptor C (CD45), increases translation in plants^32^. CBM3 tightly binds to microcrystalline cellulose (MCC) beads. The SUMO domain of *Brachypodium* is used to release the C-terminal region containing CES3 from the full-length recombinant protein by proteolysis, using bdSNEP1, a SUMO protease from *Brachypodium*^33^. The His-tag of 6 histidine residues (His) was used for western blot analysis and purification using the Ni^2+^-NTA affinity column. The ER retention signal HDEL leads to a high level of protein accumulation in the ER. To express the recombinant protein BMCS:CES3:His, the recombinant gene construct *BMCS:CES3:His* was fused with a 5’ UTR sequence showing high translational efficiency^32^ and then placed between the CaMV 35S promoter and the Hsp terminator^31^.

To express the recombinant protein (BMCS:CES3:His) in *N*. *benthamiana*, *BMCS:CES3:His* was introduced into the *Agrobacterium* strain GV3101. Subsequently, *Agrobacterium* harbouring *BMCS:CES3:His* and *Agrobacterium* harbouring gene silencing suppressor, *p38*, were mixed at 1:1 and used for syringe-mediated infiltration into *N*. *benthamiana*. Leaf tissues were harvested at 3-, 5-, or 7-days post-infiltration (dpi). Total extracts were prepared from leaf tissues and fractionated into soluble and pellet fractions. These fractions were separated by SDS-PAGE and analysed by western blotting using an anti-CBM3 antibody. The protein level of BMCS:CES3:His detected at the position of approximately 77 kD gradually increased with time (Fig. 1B) (a full version of modified immunoblot and CBB staining membranes are available in supplementary Figs. S1–S4). Co-expression of *p38* was crucial to increasing the expression level, as reported previously^34,35^. However, most proteins were detected in the pellet fraction at 5 and 7 dpi, indicating that recombinant proteins exist largely as protein aggregates. To improve the solubility of CES3-containing recombinant proteins, we used the conserved hydrophilic N-terminal (NT) domain of MaSp 1b, a major ampullate spidroin 1B, from *Nephila clavipes*. The NT domain of MaSp 1a of *Euprosthenops australis* helps to sequester the hydrophobic portion of spidroin from aqueous surroundings by forming a micellar structure^36^. A previous study showed that the substitution of Asp40 with Lys and Lys65 with Asp in the NT domain of MaSp 1a of *Euprosthenops australis* prevents dimer formation via decreased electrostatic interaction between monomers. However, the mutant NT domain retained its ability to solubilise a neighbouring hydrophobic domain by micelle formation at a wide range of pH^37^. We also substituted Asp59 with Lys and Lys84 with Asp to produce NT-m. We replaced the M domain in *BMCS:CES3:His:HDEL* with the NT-m domain to generate *BiP:NT-m:CBM3:SUMO:CES3:His:HDEL (BNCS:CES3:His)* (Fig. 1A). *Agrobacterium* harbouring *BNCS:CES3:His* infiltrated *N*. *benthamiana* leaf tissues with or without *Agrobacterium* harbouring *p38*. Total extracts were prepared from leaf tissues that had been collected at 3, 5, and 7 dpi and separated into soluble and insoluble fractions. These fractions were separated by SDS-PAGE and analysed by western blotting against the anti-CBM3 antibody. The expression level increased with time, and *p38* was critical for high-level expression. Moreover, recombinant protein BNCS:CES3:His at a position of approximately 78 kD was largely detected in the soluble fraction (Fig. 1C), indicating that the NT-m domain leads to the solubilisation of BNCS:CES3:His (Fig. 1C). However, the anti-CBM3 antibody detected two bands at positions 78 and 51 kD, indicating that proteolytic processing occurs in the recombinant protein in vivo. The upper band corresponds to the full-length recombinant protein. A previous study showed that a recombinant protein containing the SUMO domain is subject to proteolytic cleavage at the C-terminal end of the SUMO domain by an unknown endogenous protease^33^. Thus, BNCS:CES3:His might have been subject to proteolysis at the C-terminal end of the SUMO domain to give a truncated polypeptide at the 51 kD position^33^.

### Purified CES3:His from protein extracts of *N. benthamiana* plants binds with *E. coli* cells

**Figure 2:**
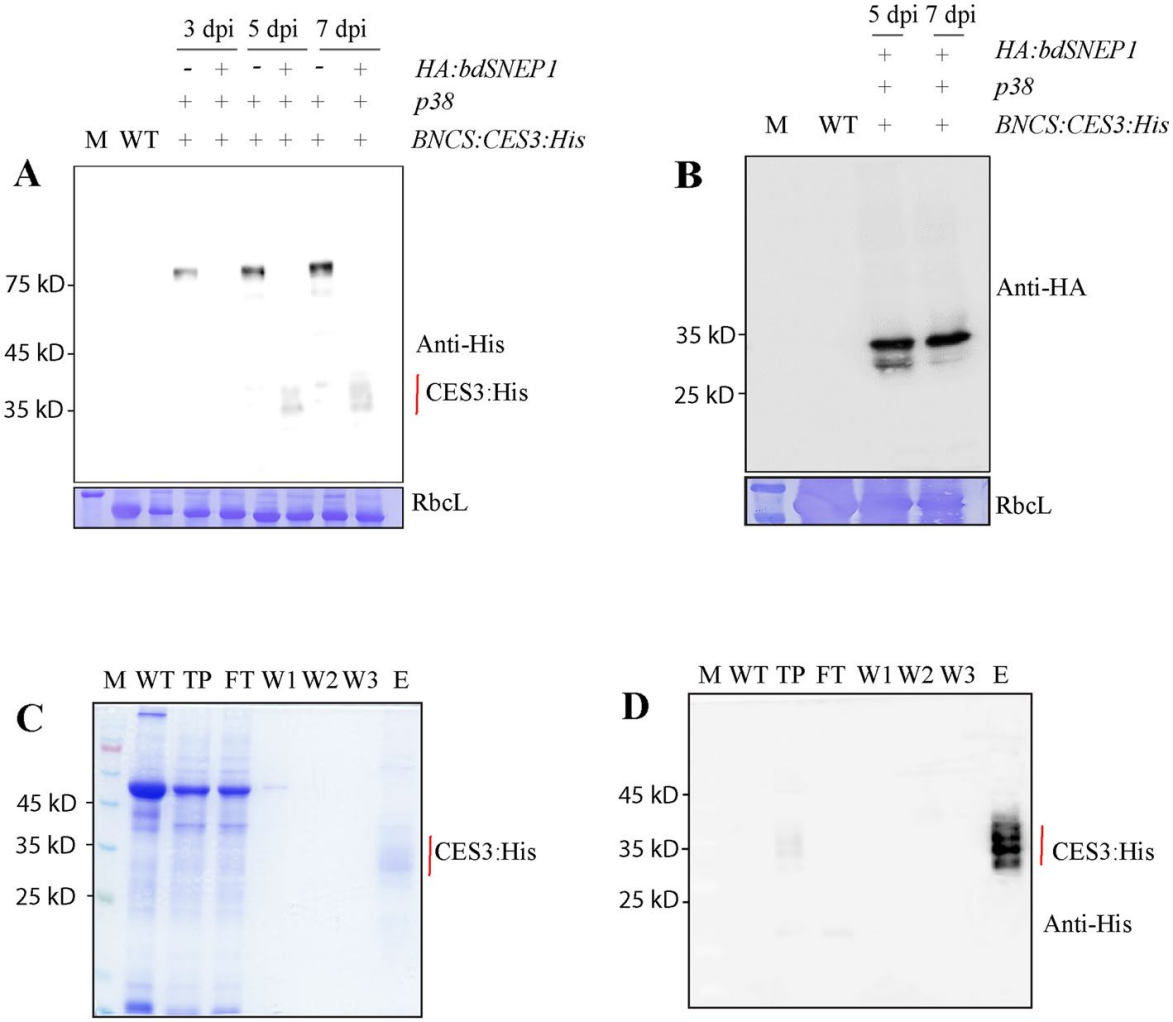
Release of CES3 from full-length recombinant proteins in vivo and its purification. (**A**, **B**) Release of CES3:His from full-length recombinant proteins by bdSENP1 in vivo. *BNCS:CES3:His* was co-expressed with *HA:bdSNEP1* and *p38*. Total protein extracts were prepared from leaf tissues harvested at different dpi, separated by SDS-PAGE, and analysed by western blotting using anti-His (**A**) or anti-HA (**B**) antibodies. (**C**, **D**) Purification of CES3:His. The total protein extracts were loaded onto a column with Ni^2^^+^-NTA-agarose beads. The Ni^2^^+^-NTA column was washed several times with buffer containing 20 mM imidazole, and proteins were eluted with buffer containing 250 mM imidazole. The samples were collected at different stages during purification. Proteins were separated by SDS-PAGE and detected by staining with Coomassie brilliant blue or analysed by western blotting using the anti-His antibody (**D**). M, Marker; TP, total protein extracts; FT, flow-through; W1, 2, and 3, washing off 1, 2, and 3; E, elution.

Next, we wanted to test whether plant-produced CES3 binds to LPS. First, we attempted to release the CES3:His region from full-length recombinant protein BNCS:CES3:His via proteolytic cleavage using HA:bdSENP1 in vivo. In a previous study, we showed that co-expression of HA:*bdSENP1* with the target protein, hLIF, leads to proteolytic processing *in vivo*^33^. Both *HA:bdSENP1* and *BNCS:CES3:His* were co-expressed in the leaf tissues of *N*. *benthamiana* via *Agrobacterium*-mediated infiltration. *p38* was also included in the expression. Total extracts from leaf tissues were analysed by western blotting using the anti-His antibody. At both 5 and 7 dpi, co-expression of *HA:bdSENP1* with *BNCS:CES3:His* led to release CES3:His which was detected at the position of approximately 28 to 36 kD. The multiple bands corresponded to the size of fully or partially N-glycosylated CES3:His (Fig. 2A). The results indicated that HA:bdSENP1 leads to the proteolysis of BNCS:CES3:His in vivo. However, the co-expression of *HA:bdSENP1* caused a great decrease in the expression of recombinant protein. We also confirmed the expression of HA:bdSNEP1, which was tagged with a small epitope, HA, at the N-terminus, by western blot analysis using an anti-HA antibody (Fig. 2B). Next, we purified CES3:His proteins using the His-tag. Protein extracts were prepared from the leaves of *N*. *benthamiana* co-infiltrated with *BNCS:CES3:His*, *HA:bdSENP1*, and *p38*, harvested at 7 dpi, and used for purification of CES3:His using Ni^2+^-NTA agarose beads. Purified CES3:His was analysed by both SDS-PAGE and western blotting against the anti-His antibody. CES3:His was detected at positions ranging from 28 to 35 kD, indicating that CES3:His is N-glycosylated (Fig. 2C,D).

**Figure 3:**
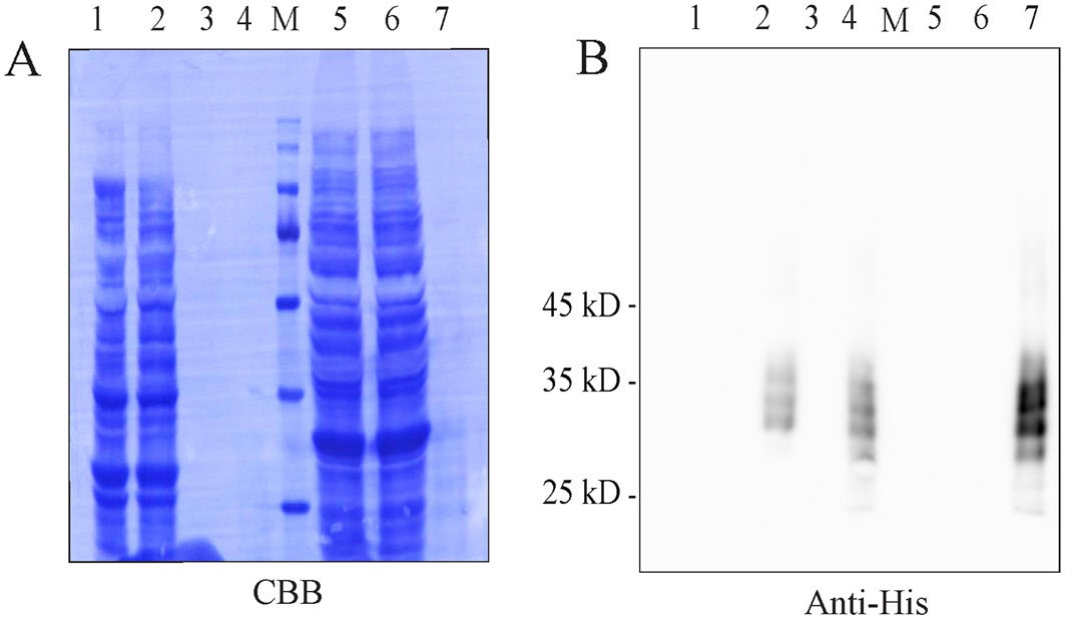
Purified CES3:His binds with *E*. *coli*. (**A**, **B**) Binding of CES3:His with *E*. *coli* and *L. sakei* cells. *E*. *coli* strain JM109 and *L. sakei* was grown in LB and MRS media, respectively overnight. The cells were collected by centrifugation and washed several times with PBS buffer. Purified CES3:His protein was incubated with *E*. *coli* and *L. sakei* cells. Cells were pelleted again by centrifugation and washed five times. The pellet fraction containing *E*. *coli* and *L. sakei* together with any bound proteins was boiled in SDS sample buffer, and proteins were separated by SDS-PAGE and stained with CBB (**A**) or analysed by western blotting using anti-His antibody (**B**). *E*. *coli* and *L. sakei* cells without protein incubation were used as a negative control. Lane 1, only *E. coli* without CES3:His treatment; lane 2, *E. coli* treated with CES3:His; lane 3, Flowthrough after binding with *E. coli*; lane 4, only CES3:His protein as control; M, Protein Marker; lane 5, only *L. sakei* without CES3:His treatment; lane 6, *L. sakei* treated with CES3:His; lane 7, Flowthrough after binding with *L. sakei.*

We determined whether plant-produced CES3 binds to LPS. Gram-negative bacteria, such as *E*. *coli*, contain LPS as a major component of the outer membrane. Therefore, we used *E*. *coli* to test the functionality of CES3:His for binding with LPS. *E*. *coli* JM109 cells grown overnight were collected by centrifugation and washed twice with PBS. Purified CES3:His was mixed with *E*. *coli* and incubated at room temperature. *E*. *coli* cells were precipitated to the pellet fraction by centrifugation and washed extensively with PBS. The pellet fraction containing *E*. *coli* was separated by SDS-PAGE and analysed by western blotting using an anti-His antibody. CES3:His at 28 to 36 kD was detected in the *E*. *coli* pellet fraction (Fig. 3A,B), indicating that CES3:His tightly binds to *E*. *coli*. *E*. *coli* cells alone and CES3:His alone were used as controls. Using the similar procedure, we also tested whether CES3-His binds to gram-positive bacteria, *Lactobacillus Sakei*, and found that CES3-His did not bind to *L. Sakei* (Fig. 3A,B).

### Fusion of cellulose-binding domain CBM3 to the C-terminus of CES3 immobilized CES3 on MCC beads

**Figure 4:**
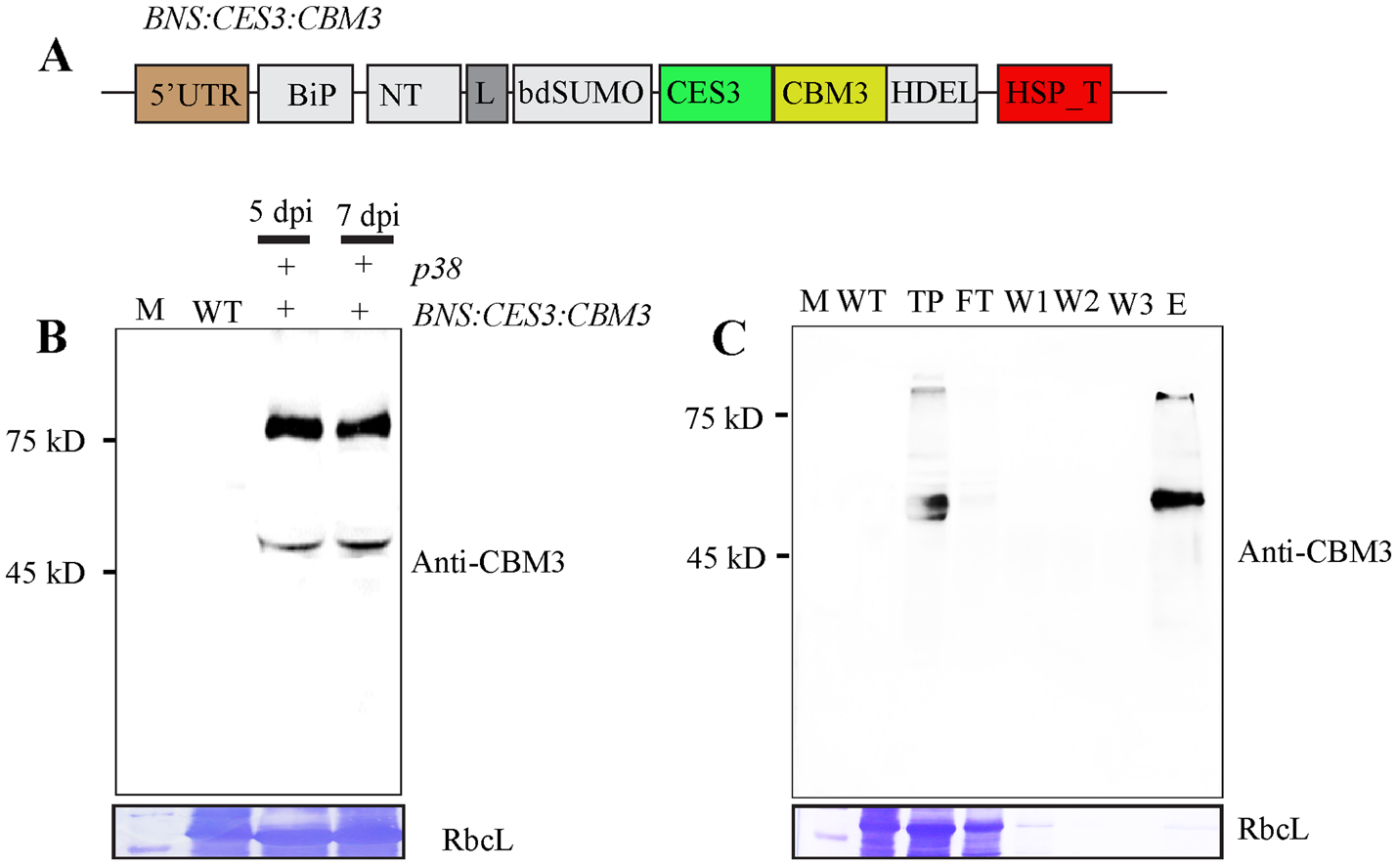
Recombinant proteins BNS:CES3:CBM3 immobilised onto MCC beads. (**A**) Schematic presentation of BNS:CES3:CBM3. (**B**) Expression of *BNS:CES3:CBM3*. *BNS:CES3:CBM3* and *p38* were introduced into *N*. *benthamiana* leaf tissues via *Agrobacterium*-mediated infiltration. Total protein extracts were prepared from leaf tissues harvested at 5 and 7 dpi and analysed by western blotting using anti-CBM3 antibody (upper panel). The membrane was stained with CBB, and RbcL was used as loading control (bottom panel). (**C**) Binding of BNS:CES3:CBM3 to MCC beads. Total protein extracts were incubated with prewashed MCC beads for 1 h. Beads were pelleted, washed, and boiled in 1X loading buffer. Proteins were separated by SDS-PAGE and analysed by western blotting using anti-CBM3 antibody. WT, non-transformed plants; FT, flow-through; W1-3, washing-off 1–3; E, MCC beads-bounded BNS:CES3:CBM3.

We evaluated whether CES3 could be used to remove endotoxins from protein samples. We designed a new recombinant construct, *BiP:NT-m:SUMO:CES3:CBM3:His:HDEL (BNS:CES3:CBM3)*, which contains the CBM3 domain at the C-terminus of CES3. In this construct, the CES3:CBM3 domain can be released from the full-length recombinant protein by bdSENP1 and can also be immobilised onto microcrystalline cellulose (MCC) beads by the CBM3 domain (Fig. 4A). *Agrobacterium* harbouring *BNS:CES3:CBM3* and *Agrobacterium* harbouring *p38* were mixed at a 1:1 ratio and used to co-infiltrate *N*. *benthamiana* leaf tissues. Total extracts were prepared from leaf tissues harvested at 5 and 7 dpi and analysed by western blotting using an anti-CBM3 antibody. BNS:CES3:CBM3 was expressed at high levels on both dpi (Fig. 4B). Next, we examined the binding of BNS:CES3:CBM3 to the MCC beads. Total soluble protein extracts were incubated with prewashed MCC beads at 4 °C with gentle shaking for 2 h. The MCC beads were collected after centrifugation and washed with buffer three times. Protein-bound MCC beads were released by boiling in SDS-PAGE sample buffer, separated by SDS-PAGE, and analysed by western blotting using an anti-CBM3 antibody. BNS:CES3:CBM3 (and CES3:CBM3) was detected in the protein sample released from the MCC beads, indicating that the recombinant proteins bind to the MCC beads (Fig. 4C). Unbound supernatant or washing-off solutions were included in the analysis. These samples did not show any signal of BNS:CES3:CBM3, indicating that BNS:CES3:CBM3 showed strong binding to the MCC beads.

### Generation of transgenic *Arabidopsis* to produce BNS:CES3:CBM3 in an endotoxin-free condition

**Figure 5:**
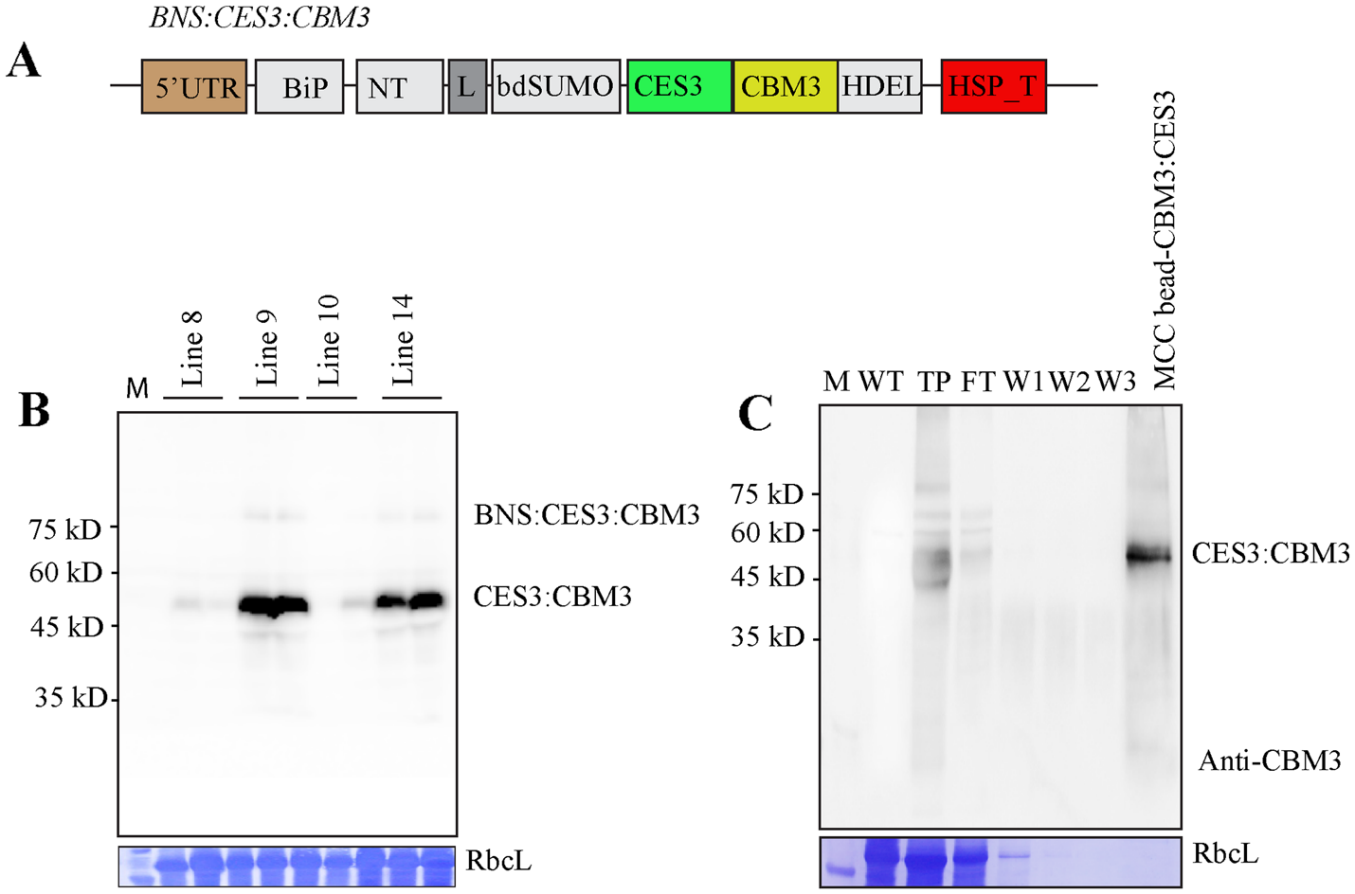
Expression of CES3-containing recombinant proteins in transgenic *Arabidopsis* plants and immobilisation of processed CES3:CBM3 onto MCC beads. (**A**) Expression of BNS:CES3:CBM3 in transgenic *Arabidopsis* plants. Total protein extracts were prepared from homozygous plants of the T3 generation and analysed by western blotting using an anti-CBM3 antibody. The number on the top indicates independent lines. (**B**) Immobilisation of processed CES3:CBM3 onto MCC beads. Transgenic *Arabidopsis* plants expressing *BNS:CES3:CBM3* were grown under endotoxin-free conditions. Total protein extracts were prepared and mixed with MCC beads that had been washed and suspended in endotoxin-free water. The mixture was incubated at room temperature for 2 h, and MCC beads were extensively washed to remove unbound proteins. The samples were collected at every step during immobilisation. Proteins were separated by SDS-PAGE and analysed by western blotting using an anti-CBM3 antibody. M, size standard; WT, non-transgenic plants; TP, total protein extracts; FT, flow-through; W1-3, washing-off solution 1–3; MCC-CES3:CBM3, MCC beads bounded with CES3:CBM3.

To further explore the possibility of developing an LPS removal system using BNS:CES3:CBM3, we sought to produce BNS:CES3:CBM3 in transgenic plants. In *N*. *benthamiana*, we used the transient expression of *BNS:CES3:CBM3:His:HDEL* via infiltration mediated by *Agrobacterium*, a Gram-negative bacterium. Thus, proteins produced in *N*. *benthamiana* might have a chance of LPS contamination. Thus, to produce BNS:CES3:CBM3 in a completely endotoxin-free environment, we generated transgenic *Arabidopsis* plants expressing *BNS:CES3:CBM3* (Fig. 5A) using the floral dipping method^38^. The expression of *BNS:CES3:CBM3* in transgenic plants was examined at the T3 generation. Total protein extracts from individual plants of the T3 generation were separated by SDS-PAGE and analysed by western blotting using an anti-CBM3 antibody. Of the many transgenic lines, independent lines 9 and 14 showed high expression (Fig. 5B). The anti-CBM3 antibody detected a strong band at 48 kD and a weak band at 78 kD (Fig. 5B). The 78 kD protein band corresponded to full-length BNS:CES3:CBM3, whereas the smaller band at 48 kD represented a truncated form corresponding to CES3:CBM3, indicating that the full-length protein undergoes proteolytic processing in vivo. One possibility is that BNS:CES3:CBM3 is cleaved at the SUMO site by an endogenous protease, even without co-expression of *bdSNEP1*. This is similar to the transient expression of BNS:CES3:CBM3 in *N*. *benthamiana*.

Next, we purified CES3:CBM3 using MCC beads. Transgenic *Arabidopsis* plants were grown in a growth chamber under aseptic conditions. Protein extracts were prepared from leaf tissues using an endotoxin-free buffer. The total soluble protein extracts were mixed with prewashed MCC beads. After incubation, the MCC beads were precipitated by centrifugation and washed extensively. Proteins released from the MCC beads by boiling were separated by SDS-PAGE and analysed by western blotting using an anti-CBM3 antibody. A protein band at the position of 48 kD was detected, indicating that the proteolytically processed form of CES3:CBM3 in vivo could be purified by MCC beads (Fig. 5C).

### CES3:CBM3 bound to MCC beads removes endotoxin from protein samples

**Figure 6:**
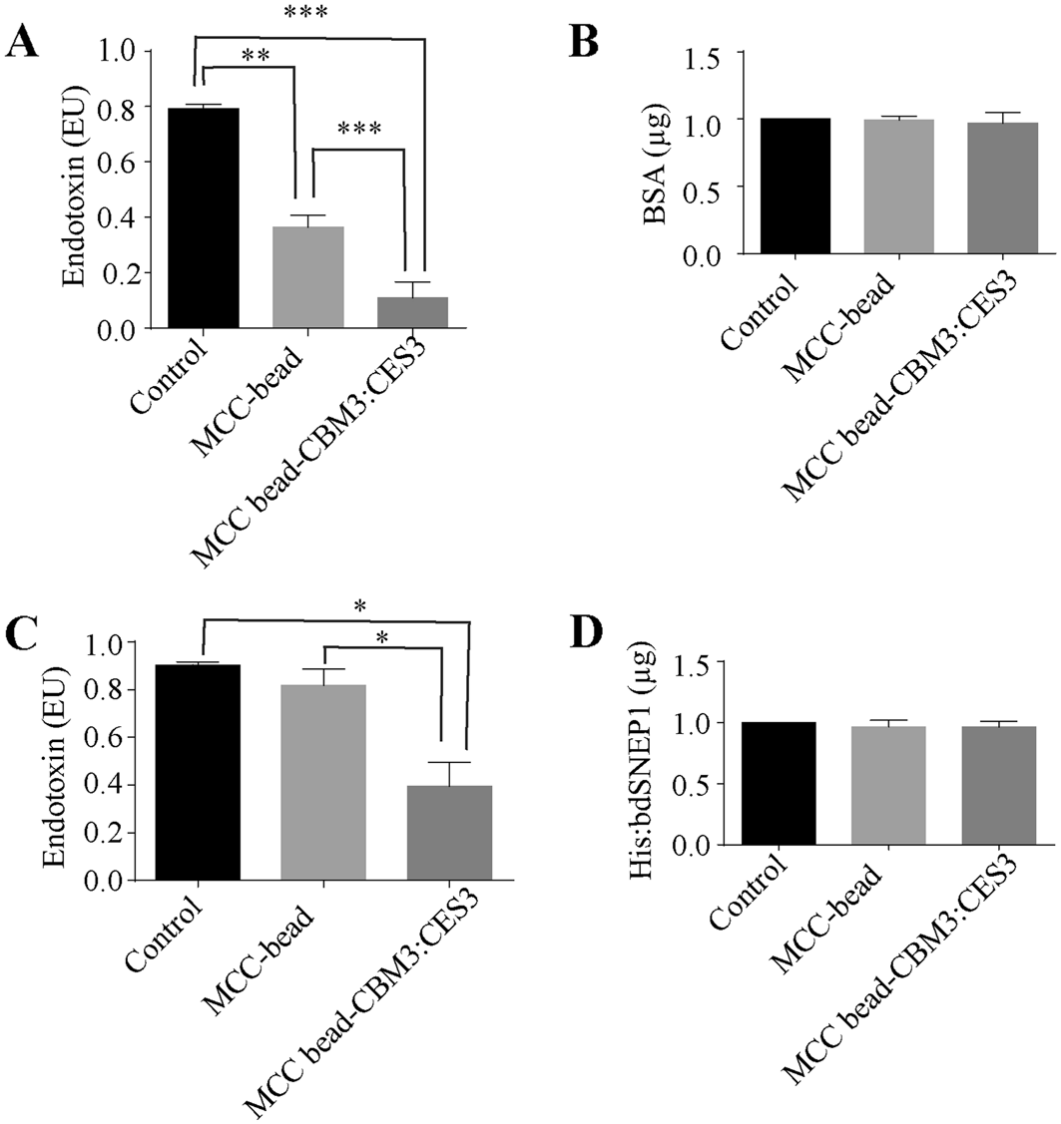
CES3:CBM immobilised onto MCC beads removes LPS from protein samples. (**A**, **B**) LPS removal from artificially constituted LPS/BSA mixture. An artificial mixture of LPS (*E*. *coli* O111:B4) and BSA was prepared by mixing 0.9 EU of LPS and 1 µg BSA in 1 mL and incubated with MCC beads only and CES3:CBM3-immobilised MCC beads for 30 min at room temperature. MCC beads were precipitated by brief centrifugation, and the MCC beads and supernatant were collected separately. The amount of LPS present in the supernatant was measured using the LAL test kit and calculated using the standard curve (**A**). The amount of BSA in the supernatant was also measured (**B**). Statistical significance was analysed using Student’s t-test in GraphPad Prism, where the error bar is ± SEM (n = 4). ***p < 0.0002 (Control *vs*. CES3:CBM3-MCC beads); **p < 0.003 (Control *vs*. MCC beads); and ***p < 0.0004 (MCC beads *vs*. CES3:CBM3-MCC beads). (**C**, **D**) LPS removal from proteins purified from *E*. *coli* extracts. His:bdSNEP1 was expressed in *E*. *coli* strain BL21 and purified using Ni^2+^-NTA. The purified His:bdSNEP1 sample was adjusted to contain approximately 1 EU LPS and 1 μg/mL protein. The effective range of the LAL test kit was less than 1 EU. Purified His:bdSNEP1 that had been contaminated with ~ 1 EU LPS was mixed with MCC beads alone or CES3:CBM3-MCC beads, and incubated for 30 min at room temperature. MCC beads were pelleted by brief centrifugation, and the supernatant was collected separately. The amount of LPS in the supernatant was measured using the LAL test kit and calculated using the standard curve (**C**). Protein amounts in the supernatant were also measured using the Bradford method (**D**). Statistical significance was analysed using Student’s t-test in GraphPad Prism, where the error bar is ± SEM (n = 3). *p < 0.01, **p < 0.003.

We examined whether CES3:CBM3 bound to MCC beads could remove LPS from the protein samples. We prepared LPS-containing protein samples by mixing 1 µg endotoxin-free BSA (bovine serum albumin) with 0.9 EU LPS of *E*. *coli* O111:B4. The endotoxin-containing BSA solution was incubated with 2 mg MCC beads alone or with CES3:CBM3-bound MCC beads for 30 min at room temperature. After centrifugation of the samples, the MCC beads and supernatant were collected separately. The supernatant was examined to determine the BSA and LPS content. The amount of LPS in the supernatant from samples incubated with CES3:CBM3-bound MCC beads and MCC beads alone was reduced to 80 and 20% of the input of LPS, respectively, indicating that CES3:CBM3 immobilised onto MCC beads can remove LPS from protein samples. In addition, MCC beads removed LPS to a certain degree (Fig. 6A). We examined the amount of BSA in the supernatant and found no loss of BSA by the MCC beads (Fig. 6B).

We further tested whether CES3:CBM3 bound to MCC beads can remove endotoxins from protein samples when proteins are produced from *E*. *coli*. We purified His:bdSNEP1 from *E*. *coli* using Ni^2+^-NTA affinity column chromatography. Approximately 1 µg of His:bdSENP1 containing approximately one EU of endotoxin was incubated with CES3:CBM3-MCC beads for 30 min at room temperature. As a control, we included MCC beads alone. After centrifugation, the supernatant was collected, and the amount of endotoxin was measured using an LAL chromogenic endotoxin measuring kit. The LPS in the supernatant from MCC beads alone and CES3:CBM3-MCC beads contained 90 and 40% of the input of LPS, respectively (Fig. 6C), indicating that CES3:CBM3-MCC beads can remove LPS from protein samples during protein purification from *E*. *coli*. We examined the amount of His:bdSENP1 in the supernatants and found that there was no loss of bdSENP1 by the MCC beads (Fig. 6D).

## Discussion

**Figure 7:**
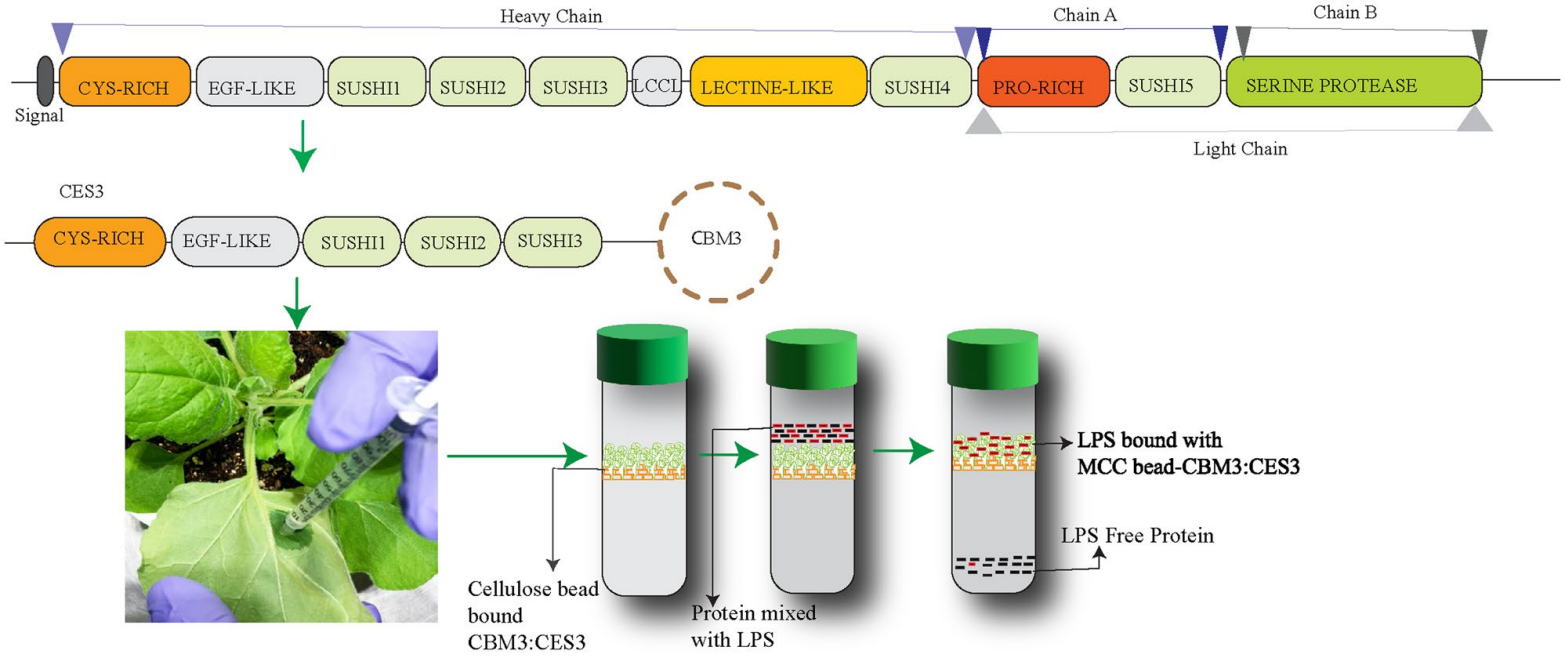
A model of LPS removal from protein samples using CES3:CBM3 immobilised onto MCC beads. Factor C of *Limulus* amoebocyte lysate (LAL) is composed of different domains, and the N-terminal CES3 is responsible for LPS sensing and shows high affinity binding. We produced a chimeric recombinant protein consisting of CES3 and CBM3, a cellulose-binding domain, in plants and immobilised onto MCC beads. The CES3:CBM3-MCC bead can be used to remove LPS from the protein samples.

In this study, we showed that CES3, the LPS-binding N-terminal region of Factor C from the horseshoe crab, can be produced as part of a recombinant protein with the ability to bind to LPS. The CES3-containing protein, CES3:CBM3, was easily purified from plant extracts using MCC beads. Moreover, the CES3 region immobilised onto MCC beads via the CBM3 of recombinant protein CES3:CBM3 removed a significant amount of endotoxin from the protein sample without affecting the amount of protein (Fig. 7).

The N-terminal region of Factor C containing cysteine-rich, EGF-like, and sushi 1–3 domains is sufficient to bind to LPS at an extremely high affinity^23–26,39^. Factor C has been reported to detect a femtogram of endotoxins, which is much lower than the detection limit recommended for pharmaceutical products^26,40^. However, it is not known which domain(s) of the N-terminal region of Factor C is responsible for LPS binding; both the cysteine-rich domain^27^ and sushi 1–3 domains^41,42^ have been reported to specifically bind to LPS. We used the N-terminal CES3 region, which contains both the cysteine-rich domain and sushi 1–3 domains for LPS binding. When we expressed CES3-containing recombinant protein in the ER, it was produced mostly as an insoluble aggregate. To solubilise CES3-containing recombinant proteins, we exploited an N-terminal domain of spidroin, which is crucial for the solubilisation of spidroin, one of the most insoluble proteins in the spinning ducts of silkworms. It has been proposed that the conserved hydrophilic N-terminal (NT) domain sequesters the hydrophobic portion of spidroin from aqueous surroundings by forming a micellar structure^36^. The NT domain is known to form a dimer^43,44^. As reported previously by Kronqvist et al. (2014)^43^, to prevent dimer formation of the NT domain, we introduced two mutations, Asp59/Lys and Lys84/Asp, to give NT-m. Fusion of NT-m greatly improved the solubility of CES3-containing recombinant protein, similar to the previous study^37^. At the same time, this brought another unexpected outcome: the processing of full-length recombinant protein into a smaller form with an apparent molecular weight of 48 kD. Based on the size and the fact that the 48 kD band is detected by the anti-CBM3 antibody, we thought that the 48 kD band corresponds to BNCS that is produced by processing downstream of the SUMO domain. In fact, a previous study in our lab showed that SUMO domain-containing recombinant proteins are processed downstream of SUMO by unknown endogenous proteases in the ER in *N*. *benthamiana*^33^. In addition, *BNS:CES3:CBM3* also produced the processed form in transgenic *Arabidopsis* plants; transgenic *Arabidopsis* plants that harbour BNS:CES3:CBM3 produced the 48 kD band as a major form and the 78 kD band as a minor form. It also appeared that the full-length protein was processed downstream of the SUMO domain to yield the 48 kD band. In fact, this is advantageous in producing CES3:CBM3 without extra domains in the N-terminal region used for recombinant protein expression, but it is not directly related to the functionality of the recombinant protein.

The most important aspect of this study was whether CES3 produced in plants maintains its ability to bind to LPS. Purified CES3 or CES3:CBM3, which had been released from full-length recombinant protein, showed a binding affinity with intact *E*. *coli* (Fig. 3A,B). Moreover, CES3:CBM3 produced in transgenic *Arabidopsis* plants and immobilised onto MCC beads was able to remove 80% of *E*. *coli* O111:B4 endotoxins contaminated with BSA without affecting the protein amount(Fig. 6A). Nevertheless, the MCC beads alone also showed a certain degree of binding to the endotoxin. In addition, CES3:CBM3 immobilised onto MCC beads was able to remove endotoxins from the recombinant proteins produced from *E*. *coli*, confirming the functionality of CES3-containing recombinant proteins in removing LPS from protein samples (Fig. 6C). In the current study, we did not directly compare the ability of our system with others in the market to remove endotoxins from protein sample^33^. On the market, however, most of the available cost-effective endotoxin removal kits are prepared based on affinity resin. These resins were conjugated with affinity ligands, such as poly(ε-lysine) and polymyxin B.

In removing endotoxins from protein samples, two important aspects of the LPS-binding ligands are how strongly and how much the ligand can bind to LPS. A previous study showed that the sushi1-3 domains show a high affinity to endotoxins, with a Kd value of 1.7 × 10^–10^ M^25^. In comparison, polymyxin B and poly(ε-lysine) had Kd values of 7.1 × 10^–7^ M and 3.6 × 10^–5^ M, respectively^45^. Thus, the sushi1-3 domains have a higher binding affinity to LPS than polymyxin B and poly(ε-lysine). The currently acceptable level of endotoxin is 0.1 ng/ml. Thus, a ligand with a Kd value of 1.7 × 10^–10^ M would be much better at removing LPS below the acceptable level. In addition to strong binding to the LPS, Factor C has also been reported to bind to other lipids including phosphatidylserine, phosphatidylinositol, and cholesterol^46^. Here we showed that CES3-His did not bind to gram-positive bacteria *L. sakei,* suggesting that the N-terminal region of Factor C, in particularly CES3, is specific to the LPS of gram-negative bacteria (Fig. 3A,B). The Factor C is a mosaic protein, composed of multiple domains. Thus, one possible explanation is that full length of Factor C is required for binding to phosphatidylserine, phosphatidylinositol, and cholesterol. It also remains to be tested how strongly Factor C binds to phosphatidylserine, phosphatidylinositol, and cholesterol in a complex situation where both LPS and these lipids are present. Another important aspect is the capacity of the ligand to remove LPS. The molecular mass of CES3:CBM3 with 48 kD is much higher than that of polymixin B and poly(ε-lysine). Thus, the capacity of CES3:CBM3-MCC beads is lower than that of polymixin B and poly(ε-lysine). However, one important aspect of LPS is that it largely exists as a micelle or aggregates in solution. Thus, CES3, which has multiple LPS-binding sites with cooperative binding between sites and a high affinity, can be better suited to remove LPS en mass in the form of micelle or aggregates, thereby compensating for the lower capacity of CES3. Another advantage of CES3:CBM3 is its ease of immobilisation onto MCC beads. There is no need to use any other means to immobilise CES3:CBM3 onto MCC beads because CBM3 shows irreversible binding to MCC beads^33^. In contrast, in the existing system, all resins, including cellulose, need to be chemically modified for the conjugation of ligands, which in turn leads to an increase in the production cost^47^. However, to upgrade this system to a commercial level requires substantial improvement in the expression level, especially in transgenic plants. In the present expression system, we found that the BNCS:CES3:CBM3 yield is around ~ 25 mg/kg (data not shown) fresh weight of biomass but it decrease significantly to ~ 10 mg/kg fresh weight (data not shown) in transgenic plants of *Arabidopsis*. In addition, *Arabidopsis* is not a suitable host for a large-scale production of recombinant proteins. One possible solution would be to generate transgenic *Nicotiana benthamiana* with more stronger expression system.

In conclusion, based on the results presented in this work, we propose that plant-produced and MCC bead-immobilised CES3:CBM3 is an effective endotoxin removal platform.

## Materials and methods

### Vector construction for transient and transgenic expression in *Nicotiana benthamiana* and *Arabidopsis*, respectively

All vectors were constructed in the backbone derived from pCAMBIA1300. The primers sequences related to this work are available in Supplementary materials (Table S1).

#### BiP:M:CBM3:SUMO:CES3:His:HDEL

The chimeric gene CES3:His:HDEL (CES3 can be found in GenBank, S77063.1, and UniprotKB, Q26422 Amino acid 26 to 301) with NaeI and Xho1 sites at the 3’ and 5’ ends, respectively, was chemically synthesised (Bioneer, Dajeon, Korea), digested with restriction endonucleases NaeI and XhoI, and ligated with p1300-C3bdSU:hIL6^31^ that had been digested with NaeI and XhoI to yield *BiP:M:CBM3:SUMO:CES3:His*:*HDEL (BMCS:CES3:His)*.

#### BiP:NT-m:CBM3:SUMO:CES3:His:HDEL

The N-terminal (459 bp) domain of MaSp1b (ACF19412.1), a major ampullate spidroin 1B, from *Nephila clavipes* was chemically synthesised with BamHI and Spe1 restriction sites at the 3’ and 5’ end, respectively (BIONEER, Korea), digested with BamHI and SpeI, and ligated to *BMCS:CES3:His:HDEL*, which had been digested with BamHI and SpeI to yield *BiP:NT-m:CBM3:SUMO:CES3:His:HDEL (BNCS:CES3:His)*.

#### BiP:NT-m:SUMO:CES3:CBM3:His:HDEL

The *CES3:CBM3* fusion construct was prepared by overlapping PCR. The *CES3* fragment was amplified by PCR using primers NaeI-CES3_F and CBM3-CES3_R, and the *CBM3* fragment using primers CES3-CBM3_F and Xho1-HDEL-CBM3_R. The two PCR products were used for overlapping PCR with Nae1-CES3_F and Xho1-HDEL-CBM3_R to produce *NaeI-CES3-CBM3-XhoI*. The PCR product and the vector *BNCS:CES3:His* were digested with NaeI and XhoI and ligated to yield *BiP:NT-m:SUMO:CES3:CBM3:His:HDEL (BNS:CES3:His)*.

### Plant material and growth

The model wild type *Nicotiana benthamiana* and Arabidopsis *thaliana* (Col0) plants were obtained in accordance with the relevant institutional, national, and international guidelines and legislation. The Arabidopsis was obtained from ABRC and the seeds of *Nicotiana benthamiana* was kindly provided by Professor Doil Choi at the Department of Plant Science, Seoul National University, Seoul, Korea. Wild-type *N*. *benthamiana* plants were grown in soil. The soil was composed of Zeolite (30 g), perlite (130 g), coco-peat (705.3 g), peat moss (130 g), and wetting agent (0.1 g). *N. benthamiana* were grown in a greenhouse under the condition of the 14 h/10 h light/dark cycle, with a light intensity of 140 µmol.m^−2^S^−1^ at 26 ± 2 °C and relative humidity of 60 ± 5% for 3–4 weeks. *Arabidopsis thaliana* was grown on growth chamber set up with 70–80 μmol.m^−2^S^−1^ white light under a 16/8 h light/dark cycle at 22 ± 1 °C.

### Transient expression of the gene in *Nicotiana benthamiana*

The expression vectors *BMCS:CES3:His and BNCS:CES3:His* were introduced into the *Agrobacterium* EHA 105 strain by electroporation and plated on LB agar (Luria Broth, Sigma-Aldrich) containing 50 µg/mL kanamycin and 50 µg/mL rifampicin. After incubation for 36 h at 26 °C, a single colony from the plate was inoculated into a 5 mL LB liquid medium containing both kanamycin and rifampicin at 50 μg/mL (AG Scientific, Pro.#R-2639) and incubated in a shaker incubator at 26 °C overnight. The culture was then inoculated into a 50 mL LB liquid medium with kanamycin and rifampicin at 50 μg/mL and incubated for 16 h. The cells were pelleted by centrifugation at 3700 × g and resuspended in infiltration buffer (10 mM MES and 10 mM Mg(SO)_4_, pH 5.6). The cell concentration was adjusted to 0.8 of OD_600_, and the cell suspension was kept at room temperature for 1 h. *Agrobacterium* harbouring *p38* was also prepared in the same way. *Agrobacterium* suspensions harbouring the target recombinant gene and *p38* were mixed at a 1:1 ratio, supplemented with 400 µM acetosyringone (Sigma, Cat. D134406) and infiltrated into leaves of 3-week-old *N*. *benthamiana* using a 1 mL syringe without needle. The infiltrated plants were placed in a greenhouse under the conditions of a 14 h light/10 h dark cycle, 25 °C temperature, 40–65% humidity, and light intensity of 140 µMm^-2^ S^-1^ for 4 to 6 weeks. The leaves were harvested on different days post-infiltration (dpi) to examine the expression of recombinant genes.

To release CES3:His from full-length BNCS:CES3:His in vivo, *Agrobacterium* harbouring *BNCS:CES3:His* was co-infiltrated with *Agrobacterium* harbouring *HA:bdSNEP1* and the gene silencing suppressor, *p38*. Thus, the concentration of Agrobacterial suspension of target construct (*BNCS:CES3:his*) was reduced to 2/3 compared with the infiltration of only *BNCS:CES3:His* and *p38* co-expression. An equal amount of total soluble protein extract was loaded on a SDS-PAGE gel to evaluate the expression level of BNCS:CES3:His and CES3:His.

### Purification of proteins using Ni^2+^-NTA affinity column chromatography and binding test with gram-positive and -negative bacteria

Infiltrated leaves were harvested at 7 dpi and ground in liquid nitrogen using a mortar and pestle. The leaf powder was mixed with 5 volumes (V/W) of extraction buffer (150 mM NaCl, 50 mM Tris–HCl, pH 7.5, 1 mM DTT, 0.1% Triton X-100, and 1X EDTA-free O’ complete protease inhibitor) and vortexed for 10 min. The mixture was centrifuged at 18,000 × g for 20 min, and the supernatant was filtrated through Miracloth (EMD Millipore Corp. Cat. 475,855) and centrifuged again at 18,000 × g for 20 min. The supernatant was filtrated through a 0.45 µM filter (GVS, Lot. 7,103,692), mixed with 10 mM imidazole (Sigma-Aldrich Lot. SLBT7469) and placed on a column packed with Ni^2+^-NTA agarose beads (QIAGEN Lot. 163013340). The protein extract was allowed to pass through the column by gravitational force, and flow-through was collected for analysis. The beads were washed with 5 column volumes of washing buffer (150 mM NaCl, 50 mM Tris–HCl, pH 7.5, and 20 mM imidazole). The protein was eluted with an elution buffer (150 mM NaCl, 50 mM Tris–HCl, pH 7.5, and 250 mM imidazole). Imidazole in the protein eluent was removed and exchanged with PBS buffer (137 mM NaCl, 2.7 mM KCl, 10 mM Na_2_HPO_4_, and 1.8 mM KH_2_PO_4_, pH 7.5) using an Amicon® Ultra centrifugal Filter and 10 kD cut-off Centricon (Merck Millipore Ltd., Lot R1CB94240).

To examine the functionality of CES3-containing recombinant proteins for binding to LPS, *E*. *coli* cells were used. *E*. *coli* strain JM109 was grown in LB medium overnight. The culture was collected by centrifugation, and the pelleted cells were resuspended in PBS buffer and washed at least 4 times using the same buffer. Purified CES3:His protein (10 µg) was mixed with 10 µL of *E*. *coli* suspension. The volume of the mixture was brought to 30 µL and incubated at room temperature for 30 min. The mixture was then centrifuged, and both the supernatant and pellet were collected separately. The pellet fraction was resuspended with 30 µL PBS buffer and 1X loading buffer, boiled for 10 min, and used as the binding fraction. The supernatant was mixed with 1X loading buffer, boiled, and used as an unbound fraction. Only *E*. *coli* cells without incubation with CES3:His were used as a negative control. Samples were separated by SDS-PAGE and analysed by western blotting using an anti-His antibody (Qiagen, Valencia, CA, USA).

To test whether the CES3-His binds to gram-positive bacteria, we grew *Lactobacillus sakei* in MRS broth medium (Sigma cat. 69,966). At 0.8 of OD_600_, the cells were harvested by centrifugation at 4000 × g for 10 min and the pellet containing *L. sakei* was washed four times with PBS. 10 µg of CES3-His protein incubated with *L. sakei* for 30 min at room temperature and centrifuged at 4.000 × g for 5 min. The pellet was collected separately and washed as described above for *E. coli*. The flowthrough, the cells after binding and the cells without incubation with CES3-His were prepared for SDS-PAGE and western blot analyses.

### Generation of transgenic *Arabidopsis* plants

To generate transgenic *Arabidopsis* plants harbouring *BNS:CES3:CBM3*, we used the floral dipping method^48^. The seeds of *Arabidopsis*, ecotype Colombia, were plated on 1/2 MS-agar plates (0.5 X Murashige and Skoog Macro and Micronutrient, 0.8% agar) (Duchefa Biocheme, Pro. No. M0222.0050), with or without 50 µg/mL hygromycin. Plants were grown on 1/2 MS plates in a growth chamber under a 16/8 h light/dark cycle, with a light intensity of 120–150 µmol.m^−2^S^−1^ at 24 °C. Transgenic plants were screened using hygromycin resistance. The expression level of *BNS:CES3:CBM3:6xHis:HDEL* was examined at the T1 generation by western blot analysis using the anti-CBM3 antibody (Bioapp, Pohang, South Korea). Homozygous plants were screened at the T2 generation and used in further analysis.

### Protein extraction, SDS-PAGE, and western blotting

Leaves harvested from both *Arabidopsis* and *N*. *benthamiana* were ground using a mortar and pestle with liquid nitrogen. The leaf powder was mixed with 2 volumes (W/V) of extraction buffer (150 mM NaCl, 50 mM Tris–HCl, pH 7.5, Triton-X-100 0.1%, DTT 1 mM, 1 X Protease inhibitor cocktail), vortexed for 5 min, and centrifuged at 1,8000 xg for 15 min. The supernatant was collected and considered total soluble protein extracts. The debris was saved and considered an insoluble protein extract. Proteins were separated by 10–12.5% sodium dodecyl sulphate–polyacrylamide gel electrophoresis (SDS-PAGE) and analysed by western blotting using appropriate antibodies. In addition, gels were stained with 0.25% CBB R-250 (AMRESCO, cat. no: 6104-59-2) in a solution containing 45% methanol and 10% glacial acetic acid. Western blot bands were visualised using an enhanced chemiluminescence kit (Amersham Pharmacia Biotech, Buckinghamshire, UK), and images were obtained using a LAS 4000 image capture system (FUJIFILM, NJ).

### Preparation of MCC and CES3:CBM3-MCC beads

Microcrystalline cellulose (MCC) beads of 50 µm in size (Sigma-Aldrich Lot. MKCB1667V) were suspended in pyrogen-free water. The suspension was shaken for a few minutes and kept on the bench for MCC beads to settle. The supernatant was discarded. This whole procedure was repeated 3 to 4 times until all dust and minute particles were removed from the MCC suspension. The MCC beads were mixed with an equal volume of endotoxin-free water and stored at 4 °C.

Just before use, the MCC beads were added with a protein extract of CBM3:CES3 and incubated for 1 h at 4 °C. The centrifuged mixture was separated from the supernatant and washed five times with washing buffer (40 mM Tris–HCl, pH 7.5).

### Endotoxin detection

The endotoxin was detected using Pierce™ LAL Chromogenic Endotoxin Quantitation Kit (Thermo Scientific, Catalogue No. 88282) following the guidelines provided by the manufacturer. Endotoxin, *E*. *coli* O111:B4, was dissolved and diluted using endotoxin-free water by following the guidelines. The endotoxin was added to 1 µg endotoxin-free BSA to a final concentration of 0.9 EU in 1 mL, vortexed, and kept at room temperature for 10 min. MCC beads and CES:CBM3 immobilised onto MCC beads were added to the mixture of BSA–endotoxin followed by incubation at room temperature for 30 min. The mixture was centrifuged briefly, and the supernatant was collected to measure the endotoxin.

We prepared a standard curve to calculate the amount of LPS in the samples. First, a serial dilution of a known amount of endotoxin was prepared according to the guidelines and used to measure the endotoxin level to generate a standard curve with a coefficient of determination (R^2^) of 0.98. The absorbance of the samples was measured using the plate reader (BioTeK) and converted to the amount of endotoxin using the standard curve.

## Supplementary Information

Supplementary Information.

## Data Availability

The source of the genetic materials such as CES3 (S77063.1), NT domain of MaSp1b (ACF19412.1), M domain from CD45 (Gene ID: 5788), CBM3 (AEI55081), bdSUMO (XP_003564931.1), BiP (AT5G28540.1) are available in NCBI (https://www.ncbi.nlm.nih.gov/). following the accession number mentioned in the bracket. All the other dataset produced in this study are included in this manuscript and supplementary information.
