# Supplementary material for: Plant produced endotoxin binding recombinant proteins effectively remove endotoxins from protein samples
Source: Sci Rep. 2022 Sep 30;12:16377. doi: 10.1038/s41598-022-20776-6 (PMC9525263; doi:10.1038/s41598-022-20776-6)
Supplement: Supplementary file 1 — Supplementary Information. [file 41598_2022_20776_MOESM1_ESM.pdf]

Supplementary Information

Plant produced endotoxin binding recombinant proteins effectively remove endotoxins from protein samples

Md Rezaul Islam Khan[1], Muthamilselvan Thangarasu[1], Hyangju Kang[2], and Inhwan Hwang[1]*

[1]Department of Life Sciences, Pohang University of Science and Technology, Pohang 37673, Korea

[2]R&D Center, BioApplications Inc., Technopark Complex, Pohang 37668, Korea

Running title: Recombinant protein for endotoxin removal

***Corresponding Author:** Dr. Inhwan Hwang, Tel. +82-54-279-2128; fax. +82-54-279-2199; Email; ihhwang@postech.ac.kr

Supporting Table S1: List of primers.

**Name of primers**	**Oligo Sequence (from 5' to 3')**
CBM3-CES3_R	CAACCCTAGATCTACTCCCTTTCCACCTTAAGGTTACCTGATAC
CES3-CBM3_F	AAGGGAGTAGATCTAGGGTTGGTATCAGGTAACCTTAAGGTGGAA
Xho1-HDEL-CBM3_R	ctcgagCTAGAGCTCATCATGGTGGTGATGATGATGGG GACCAGGTTCCTTTCCCCACACTAGAAC
NaeI-CES3_F	gccggcGGTAAGGGAGTAGATCTAGGGTTGTGTGATGAA

N.B the small letter underlined indicate restriction site.

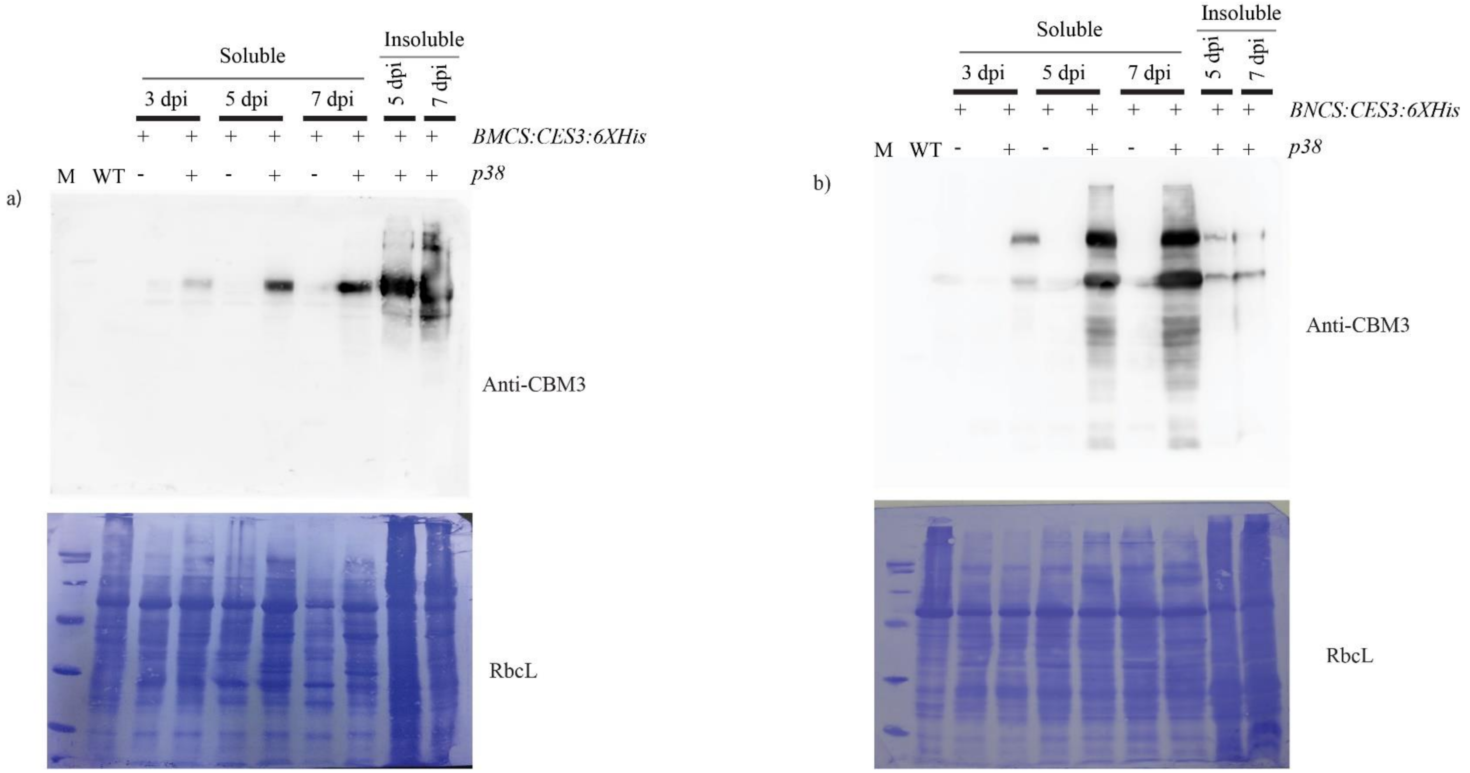

Supplementary Figure S1. Full length immunoblots for Figure 1; (a) for Figure B , (b) for Figure C.

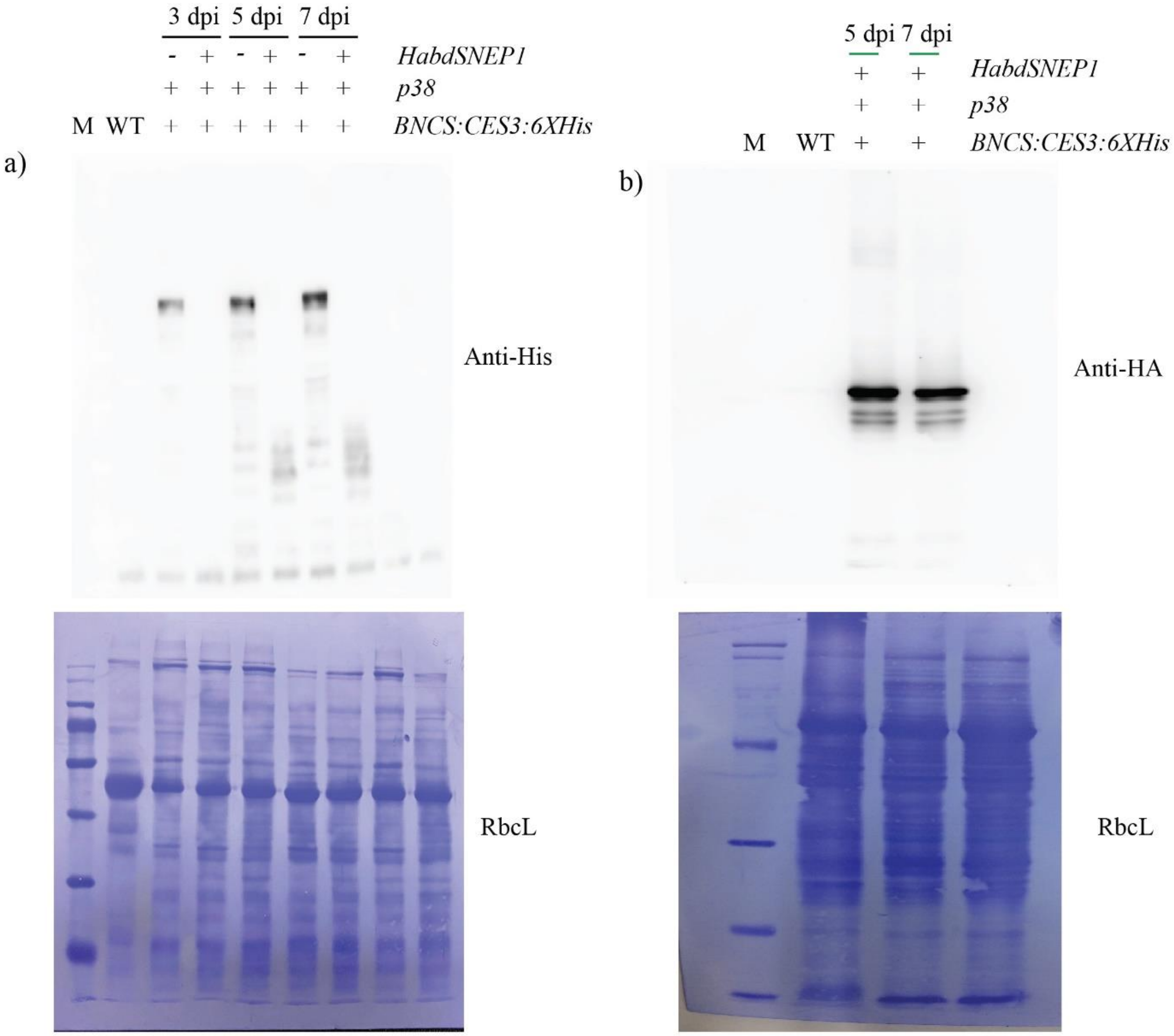

Supplementary Figure S2. Full immunoblot for Figure 2; (a) for Figure 2A and (b) for Figure 2B.

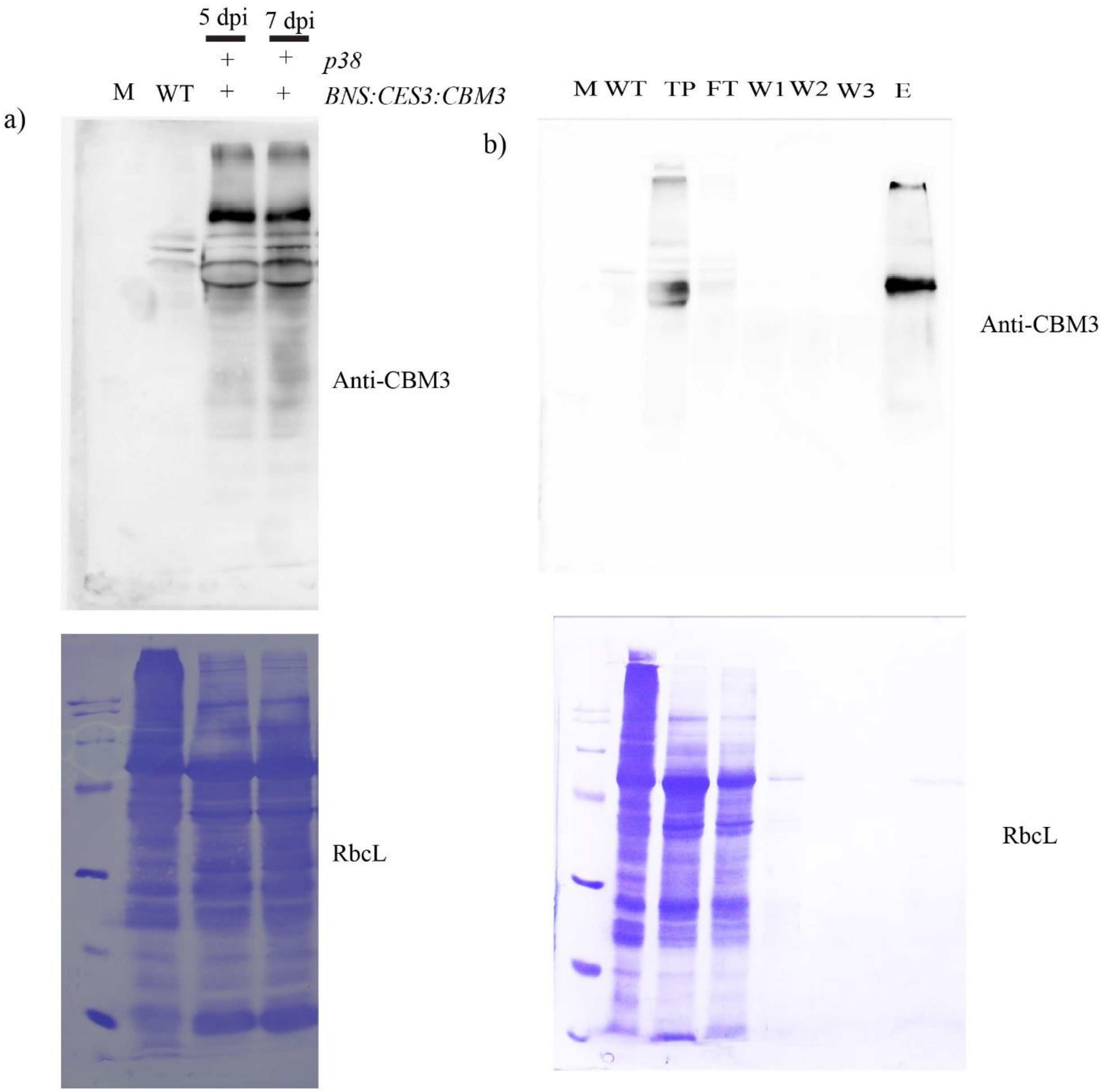

Supplementary Figure S3. Full immunoblot and CBB staining membrane for Figure 4:
(a) for Figure 4B and (b) for Figure 4C

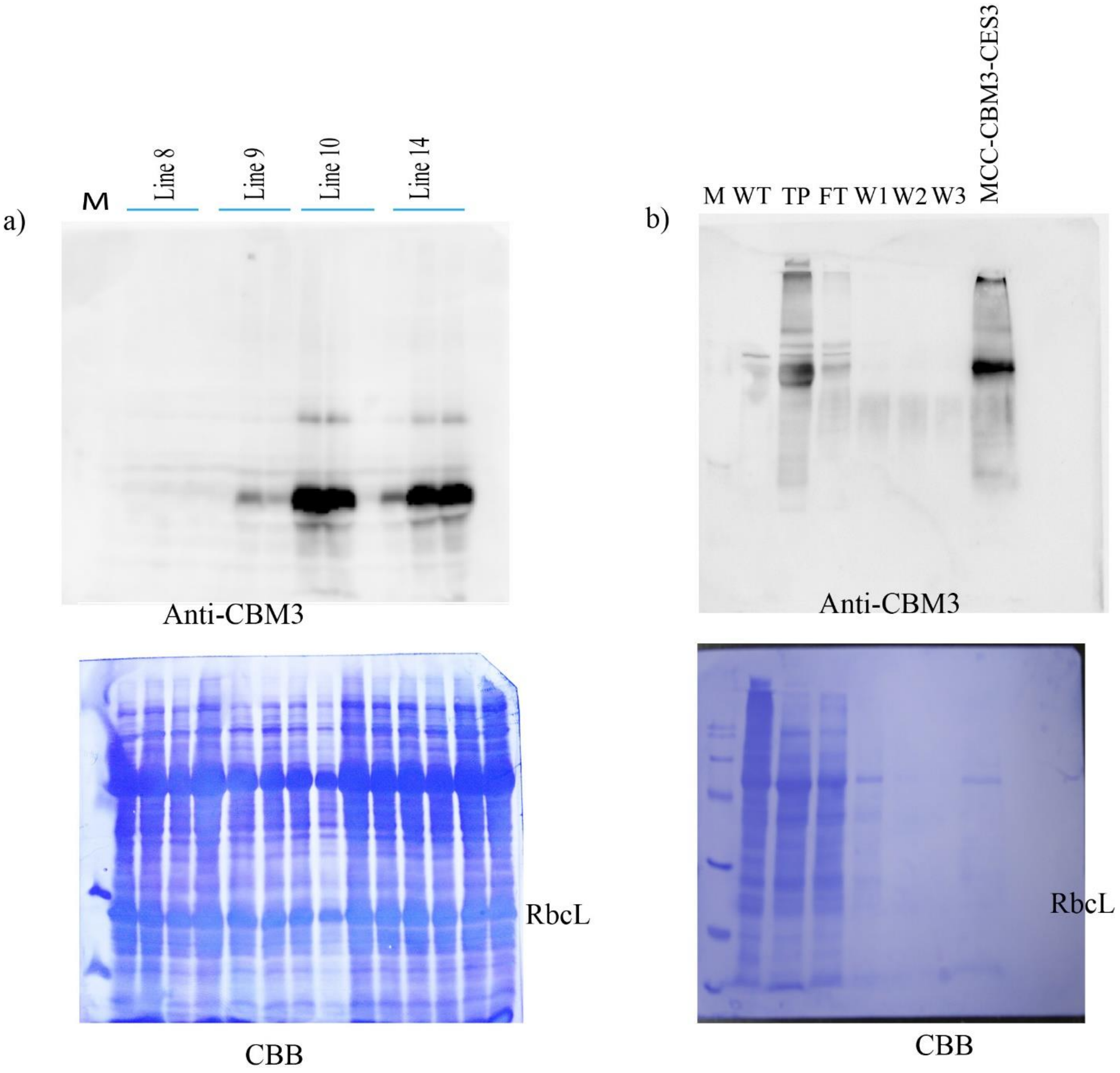

Supplementary Figure S4. Full Immunoblot and CBB staining of membrane where (a) is for Figure 5B and (b) is for Figure 5C.
